# Endothelial Heme Dynamics Drive Cancer Cell Metabolism by Shaping the Tumor Microenvironment

**DOI:** 10.3390/biomedicines9111557

**Published:** 2021-10-28

**Authors:** Sara Petrillo, Francesco De Giorgio, Joanna Kopecka, Tullio Genova, Veronica Fiorito, Anna Lucia Allocco, Francesca Bertino, Deborah Chiabrando, Federico Mussano, Fiorella Altruda, Luca Munaron, Chiara Riganti, Emanuela Tolosano

**Affiliations:** 1 Molecular Biotechnology Center (MBC), Department of Molecular Biotechnology and Health Sciences, University of Torino, 10126 Torino, Italy; francesco.degiorg@edu.unito.it (F.D.G.); veronica.fiorito@unito.it (V.F.); annalucia.allocco@unito.it (A.L.A.); francesca.bertino@unito.it (F.B.); deborah.chiabrando@unito.it (D.C.); fiorella.altruda@unito.it (F.A.); emanuela.tolosano@unito.it (E.T.); 2 Department of Oncology, University of Torino, 10126 Torino, Italy; joanna.kopecka@unito.it (J.K.); chiara.riganti@unito.it (C.R.); 3 Department of Life Sciences and Systems Biology, University of Torino, 10123 Torino, Italy; tullio.genova@unito.it (T.G.); luca.munaron@unito.it (L.M.); 4 Department of Surgical Sciences, C.I.R. Dental School, University of Torino, 10126 Torino, Italy; federico.mussano@unito.it

**Keywords:** cancer cell metabolism, tumor microenvironment, endothelial cell metabolism, tumor endothelial cells, heme metabolism, FLVCR1a, ketone bodies

## Abstract

The crosstalk among cancer cells (CCs) and stromal cells within the tumor microenvironment (TME) has a prominent role in cancer progression. The significance of endothelial cells (ECs) in this scenario relies on multiple vascular functions. By forming new blood vessels, ECs support tumor growth. In addition to their angiogenic properties, tumor-associated ECs (TECs) establish a unique vascular niche that actively modulates cancer development by shuttling a selected pattern of factors and metabolites to the CC. The profile of secreted metabolites is strictly dependent on the metabolic status of the cell, which is markedly perturbed in TECs. Recent evidence highlights the involvement of heme metabolism in the regulation of energy metabolism in TECs. The present study shows that interfering with endothelial heme metabolism by targeting the cell membrane heme exporter Feline Leukemia Virus subgroup C Receptor 1a (FLVCR1a) in TECs, resulted in enhanced fatty acid oxidation (FAO). Moreover, FAO-derived acetyl-CoA was partly consumed through ketogenesis, resulting in ketone bodies (KBs) accumulation in FLVCR1a-deficient TECs. Finally, the results from this study also demonstrate that TECs-derived KBs can be secreted in the extracellular environment, inducing a metabolic rewiring in the CC. Taken together, these data may contribute to finding new metabolic vulnerabilities for cancer therapy.

## 1. Introduction

The tumor microenvironment (TME) is a dynamic and complex niche consisting of both cancer cells (CCs) and supporting stromal cells [1]. Among them, endothelial cells (ECs) are critical determinants of tumor progression and immune evasion [2]. ECs’ contribution to tumor development does not only rely on the formation of new blood vessels (angiogenic switch). Indeed, vascular networks are not just passive conduits, and ECs supply a specific pattern of membrane-bound and secreted elements to the surrounding cells (angiocrine switch), thus actively contributing to establishing a unique pro-tumorigenic milieu [3,4].

Accumulating evidence indicates that metabolic cooperation exists between CCs and stromal cells, the latter having a crucial role in shaping CC metabolism by shuttling metabolic intermediates to support CC proliferation and survival [5]. Metabolic reprogramming is indeed a hallmark of CCs [6]. Nevertheless, ECs metabolism is markedly perturbed in cancer, too [7,8]. Tumor-associated ECs (TECs) are indeed activated ECs, which undergo a metabolic rewiring to sustain neo-angiogenesis, the so-called “angiogenic metabolic switch” [9]. However, changes in EC metabolism also affect the array of soluble factors and metabolites that are released in the TME, thus affecting CC metabolism. In this scenario, CCs and TECs establish a vicious cycle characterized by the bidirectional transfer of a complex network of signals and organic compounds. Targeting the metabolic interplay within the TME has gained attention as a promising strategy in cancer treatment [10].

Heme, the complex of iron and porphyrin IX, is an essential molecule with an increasingly recognized role in cell energetic metabolism. Heme homeostasis relies on the coordinated activity of enzymes and transporters that modulate heme synthesis, import/export, catabolism, or utilization [11,12,13,14,15]. In this context, heme synthesis and heme export have been shown to be co-regulated processes and constitute a unique functional axis interlinked with pathways related to cell energy production [16]. Heme synthesis starts in mitochondria with the condensation of succinyl-CoA and glycine, a reaction catalyzed by 5-aminolevulinate synthase 1 (ALAS1), the rate-limiting enzyme of the biosynthetic process. Heme export is mainly controlled by the cell membrane transporter Feline Leukemia Virus subgroup C Receptor 1a (FLVCR1a). Interestingly, FLVCR1a inhibition in CCs or TECs leads to ALAS1 inhibition, thus resulting in reduced heme synthesis [16]. These alterations in heme metabolism, in turn, promote the tricarboxylic acid (TCA) cycle flux and the oxidative phosphorylation (OXPHOS) in proliferating cells as CCs and TECs [16].

Here, we show that FLVCR1a is overexpressed in TECs, both in vitro and in vivo. Heme metabolism disruption in TEC by targeting FLVCR1a leads to enhanced fatty acid oxidation (FAO) and accumulation of ketone bodies (KBs), a likely side-product of fatty acids (FAs) breakdown. These metabolic alterations, in turn, modify the pattern of EC-derived metabolites, with a high release of KBs in the extracellular environment. Finally, our data indicate that elevated levels of KBs within the TME are sufficient to promote oxidative metabolism in the CC.

## 2. Materials and Methods

### 2.1. Cell Cultures

Human umbilical endothelial cells (HUVECs) were propagated in M199 medium (Invitrogen) with 20% heat-inactivated low-endotoxin fetal bovine serum (FBS; Gibco by Thermo Fisher Scientific, Waltham, MA, USA, catalog n° 10270106), 100 U/mL penicillin, 100 μg/mL streptomycin, 20 U/mL Heparin sodium salt from porcine intestinal mucosa (Sigma-Aldrich, St. Louis, MO, USA), and 10 ng/mL recombinant human FGF—basic (PeproTech, Hong Kong, China). HUVECs were used up to passage 6. Human adult dermal microvascular endothelial cells (HMECs) were purchased by Lonza and propagated in EndoGRO-MV-VEGF Complete Culture Media Kit (SCME003 Merck Millipore, Burlington, MA, USA) and used up to passage 12. Breast-tumor-derived endothelial cells (BTEC) and renal-tumor-derived endothelial cells (RTEC) from human breast lobular-infiltrating carcinoma biopsy and renal carcinoma, respectively, were isolated and characterized in the laboratory of Professor Benedetta Bussolati, Department of Molecular Biotechnology and Health Sciences, University of Torino, Torino, Italy [17,18]. BTECs and RTECs were maintained in EndoGRO-MV-VEGF Complete Culture Media Kit (SCME003 Merck Millipore, Burlington, MA, USA). Lewis lung carcinoma LL/2 (LLC1) cells (ATCC: CRL-1642) were cultured in Dulbecco’s modified Eagle’s medium (DMEM, high glucose, GlutaMAX supplement; Gibco by Thermo Fisher Scientific, Waltham, MA, USA, catalog n° 61965059) supplemented with 10% heat-inactivated low-endotoxin fetal bovine serum (FBS; Gibco by Thermo Fisher Scientific, Waltham, MA, USA, catalog n° 10270106). All cell media were ordinarily supplemented with antibiotics (100 μg/mL penicillin and 100 ug/mL streptomycin; Gibco by Thermo Fisher Scientific, Waltham, MA, USA, catalog n° 15140122). Cells were maintained in a 37 °C and 5% CO_2_ air incubator and routinely screened for absence of mycoplasma contamination.

### 2.2. Reagents and Materials

BTECs were treated with 500 uM trimetazidine (1-(2,3,4-Trimethoxybenzyl) piperazine dihydrochloride, 653322 Sigma-Aldrich, St. Louis, MO, USA) dissolved in Phosphate-Buffered Saline (PBS) 1× solution for 72 h. Afterward, the conditioned medium (CM) and cell lysate were collected and used for metabolic analysis or in LLC cells treatment. LLC cells were treated with increasing concentrations (i.e., 1, 5, 10 mM) of β-hydroxybutyrate (H6501 Sigma-Aldrich, St. Louis, MO, USA) dissolved in PBS 1× solution.

### 2.3. FLVCR1a Gene Silencing

*FLVCR1a* silencing was performed as described in [19]. Briefly, an shRNA targeting the sequence correspondent to the first exon of the human FLVCR1 gene was used to specifically down-regulate FLVCR1a (TRC Lentiviral pLKO.1 Human FLVCR1 shRNA set RHS4533-EG28982, clone TRCN0000059599; Dharmacon, Lafayette, CO, USA) in BTECs. For control cells, a pLKO.1 lentiviral vector expressing a scramble (scr) shRNA was used. The lentiviruses were produced in HEK293FT cells. Cells were infected with the lentiviruses in the presence of Sequabrene™ (S2667 Sigma-Aldrich, St. Louis, MO, USA). Following lentiviral transduction, cells were maintained in selective medium containing 0.002 mg/mL puromycin (puromycin dihydrochloride from Streptomyces alboniger, Sigma-Aldrich, St. Louis, MO, USA, catalog n° P8833).

### 2.4. Conditioned Medium (CM) Preparation

*FLVCR1a*-silenced and control BTECs (4–5 × 10^5^) were seeded in 10 mm tissue-culture-treated dishes in the presence of 6 mL of EndoGRO-MV-VEGF Complete Culture Media. After 72 h, BTEC-derived CM was collected and centrifugated at 300× *g* at 4 °C to eliminate cell debris. Afterward, CM was diluted 1:3 with complete DMEM culture medium. The obtained diluted CM was used to treat LLC cells for 24 h.

### 2.5. Animals

Tamoxifen-inducible, endothelial-specific *Flvcr1a*-null mice (*Flvcr1a*^iEC-KO^) were generated in our laboratory. Briefly, previously generated *Flvcr1a*^flox/flox^ mice were crossed with Cdh5(PAC)-Cre^ERT2^ mice (Tg (Cdh5-cre/ERT2)1Rha, kindly provided by Ralf H. Adams) on a C57BL/6 background. Mice were genotyped by polymerase chain reaction (PCR) analyses on genomic DNA from tail biopsies. To detect the Cdh5-Cre allele, primers Cre-Fw (5′-ACACCTGCTACCATATCATCCTAC-3′) and Cre-Rev (5′-CATCGACCGGTAATGCAG-3′) were used. To analyze the LoxP sites on Flvcr1 gene, primers ILox-Fw (5′-TCTAAGGCCCAGTAGGACCC-3′) and ILox-Rev (5′-GAAAGCATTTCCGTCCGCCC-3′) were used, given a 280 bp band for the floxed allele and a 242 bp band for the wild-type allele. To inactivate *Flvcr1a* selectively in endothelial cells, 4–6 weeks old *Flvcr1a*^flox/flox^; Cdh5-Cre^ERT2^ mice were treated intraperitoneally with 1 mg/day tamoxifen (Sigma-Aldrich, St. Louis, MO, USA, catalog n° T5648) for 5 consecutive days, followed by 1 additional day after a 2-day treatment-free interval. To detect the *Flvcr1a*-null allele resulting from Cdh5-Cre activity, primers ILox-Fw (5′-TCTAAGGCCCAGTAGGACCC-3′) and IILox-Rev (5′-AGAGGGCAACCTCGGTGTCC-3′) were used, given a 320 bp fragment. Tamoxifen-treated *Flvcr1a*^flox/flox^ mice were used as controls. All the mice were provided with food and water ad libitum. All experiments with animals were approved by the Italian Ministry of Health (562/2018-PR, 20 July 2018).

### 2.6. Xenograft Tumor Model

For the LLC xenograft model, 5 × 10^5^ LL/2 (LLC1) murine cells suspended in 100 μL PBS were injected subcutaneously into the flanks of immunocompetent syngeneic C57BL/6 mice. For tumor induction in *Flvcr1a*^iEC-KO^ mice and controls, mice were treated intraperitoneally with tamoxifen (Sigma-Aldrich, St. Louis, MO, USA; 1 mg/day for 5 consecutive days and 1 additional day after a 2-day treatment-free interval) one week before LLC cells injection.

### 2.7. Isolation of TEC and CC from LLC-Xenografts

Tumor-associated endothelial cells (TECs) and cancer cells (CCs) were isolated from LLC subcutaneous tumors developed in tamoxifen-inducible, endothelial-specific *Flvcr1a*-null mice (*Flvcr1a*^iEC-KO^) and controls. Briefly, tumors were dissected and minced into 1–2 mm fragments with a scalpel. Tissue pieces were incubated at 37 °C for 60 min in 10 mL of pre-warmed Dulbecco’s Phosphate Buffered Saline (DPBS) with calcium and magnesium (Lonza Pharma & Biotech, Basel, Switzerland, catalog n. BE17-513F) and 2 mg/mL collagenase (collagenase from Clostridium histolyticum, Type I, Sigma-Aldrich, St. Louis, MO, USA, catalog n. C0130), with regular shacking until a single cell suspension was obtained. During this incubation, the cells were mechanically dissociated at 10 min intervals by pipetting. To stop the collagenase activity, DMEM (GIBCO by Thermo Fisher Scientific, Waltham, MA, USA, catalog n. 61965059) containing 10% FBS (GIBCO by Thermo Fisher Scientific, Waltham, MA, USA, catalog n. 10270106) was added to the cell suspension, gently pelleted, and rinsed with PBS. The cells in PBS were then filtered through a 40 mm cell strainer (Corning Life Sciences, Corning, NY, USA, catalog n. 352340). Single-cell suspension was centrifuged at 300× *g* for 10 min, and TECs/CCs were isolated through MACS Technology by using nano-sized MicroBeads, following the manufacturer’s instructions. Particularly, a negative selection was performed using CD45 MicroBeads (Miltenyi Biotec, Bergisch Gladbach, Germany, catalog n. 130-052-301). CD45-negative cell fraction was then pelleted and incubated with CD31 MicroBeads (Miltenyi Biotec, Bergisch Gladbach, Germany, catalog n. 130-097-418) to isolate TECs (CD45−/CD31+ cell fraction). CCs resulted from the CD45−/CD31− cell fraction.

### 2.8. RNA Extraction and Quantitative Real-Time PCR Analysis

RNA extraction and quantitative real-time PCR (qRT-PCR) analyses were performed as described previously [19]. Briefly, total RNA was extracted using PureLink RNA Mini Kit (Thermo Fisher Scientific, Waltham, MA, USA), and 0.5–1 μg of total RNA was transcribed into complementary DNA (cDNA) by High-Capacity cDNA Reverse Transcription Kit (Thermo Fisher Scientific, Waltham, MA, USA). qRT-PCR was performed using gene-specific TaqMan™ Gene Expression Assays (Thermo Fisher Scientific Waltham, MA, USA). To detect *FLVCR1a* expression, specific primers and probes were designed using Primer Express Software Version 3.0 (Thermo Fisher Scientific, Waltham, MA, USA). qRT-PCR was performed on a 7900HT Fast or QuantStudio™ 6 Flex Real-Time PCR System (Thermo Fisher Scientific, Waltham, MA, USA), and the analyses were completed using RQ Manager or QuantStudio Real-Time PCR software. Transcript abundance, normalized to 18 s messenger ribonucleic acid (mRNA) expression, is expressed as a fold change over a calibrator sample.

### 2.9. Western Blot Analysis

To assess *FLVCR1a* expression, BTECs were lysed by rotation for 30 min at 4 °C in RIPA buffer (150 mM NaCl, 50 mM Tris-HCl pH 7.5, 1% Triton X-100, 0.5% sodium deoxycholate, 0.1% SDS, 1 mM EDTA). The buffer was freshly supplemented with 1 mM phosphatase inhibitor cocktail (Sigma-Aldrich, St. Louis, MO, USA, catalog n° P0044), 1 mM PMSF (Sigma-Aldrich, St. Louis, MO, USA, catalog n° 93482-50ML-F), and protease-inhibitor cocktail (La Roche, Basel, Switzerland, catalog n° 04693116001). The cell lysate was clarified by centrifugation for 10 min at 4 °C. Protein concentration in the supernatant was assessed by Bradford assay. For FLVCR1a protein detection, 10 μg of protein extracts were incubated 10 min at 37 °C with 1 μL of PNGase-F from Elizabethkingia meningoseptica (Sigma-Aldrich, St. Louis, MO, USA, catalog n° P-7367) to remove protein glycosylation. Before loading on 4–15% mini-PROTEAN TGX precast gel (Bio-Rad, Hercules, CA, USA, catalog n° 4568084), samples were incubated 5 min at 37 °C (FLVCR1) or 10 min at 95 °C (Vinculin, Actin-β) in 4× Laemmli buffer freshly supplemented with 8% 2-mercaptoethanol. The primary antibodies and dilutions are as follows: FLVCR1 (C-4) (Santa Cruz Biotechnology, Dallas, TX, USA, catalog n° sc-390100; 1:500), Vinculin (homemade, 1:8000), and Actin-β (Invitrogen, catalog n° MA5-32540; 1:1000). The revelation was assessed using the ChemiDoc Imaging System (Bio-Rad, Hercules, CA, USA).

### 2.10. Fatty Acids β-Oxidation Measurement

Cells were washed with fresh medium, detached with trypsin/EDTA, re-suspended at 1 × 10^5^ cells/mL in 0.2 mL of 100 mM Tris 10 mM/EDTA, and 50 µL aliquots were sonicated and used for protein measurements and normalization using the BCA1 kit (Sigma-Aldrich, St. Louis, MO, USA). The remaining samples were centrifuged at 13,000× *g* for 5 min at room temperature and re-suspended in 0.5 mL Hepes 20 mM (pH 7.4), containing 0.24 mM fatty acid-free BSA, 0.5 mM l-carnitine, 2 µCi [1-^14^C]palmitic acid (3.3 mCi/mmol, PerkinElmer, Waltham, MA, USA) and transferred into test tubes that were tightly sealed with rubber caps. In each experimental set, cells were pre-incubated for 30 min with the carnitine palmitoyltransferase inhibitor etomoxir (1 µM) or with the AMP-kinase activator 5-aminoimidazole-4-carboxamide ribonucleotide AICAR (1 mM), as negative and positive controls, respectively. After 2 h incubation at 37 °C, 0.3 mL of a 1:1 *v*/*v* phenylethylamine/methanol solution was added to each sample using a syringe, followed by 0.3 mL 0.8 N HClO_4_. Samples were incubated for a further 1 h at room temperature, then centrifuged at 13,000× *g* for 10 min. Both the supernatants, containing ^14^CO_2_, and the precipitates, containing ^14^C-acid-soluble metabolites (ASM), i.e., the main products of fatty acid β-oxidation, were collected. The radioactivity of each supernatant and precipitate was counted by liquid scintillation, according to [20].

### 2.11. Ketone Bodies Measurement

The amount of β-hydroxybutyrate, taken as an index of ketone bodies (KBs) amount, was assessed in whole-cell lysate as well as in conditioned medium using the β-hydroxybutyrate Colorimetric Assay Kit (Cayman Chemical, Ann Arbor, MI, USA), as per manufacturer’s instructions. Results were expressed as µmol/mg cellular proteins.

### 2.12. Evaluation of Ketone Bodies Flux

BTEC cells were incubated 24 h with 1 µCi [^3^H]-acetate (3.6 mCi/mmol, PerkinElmer), then washed five times with PBS, and let grow for additional 48 h in fresh medium. After 72 h of radiolabeling, both cells and medium were collected. One aliquot of medium was used to generate the BTEC CM and incubated 24 h on LLC cells; the remaining part was used for the measurement of acetoacetate, as reported [21]. BTEC cells and LLC cells were collected by gentle scraping, re-suspended in PBS, and sonicated. A total of 100 µL cell lysates was used to measure the protein amount. A total of 300 µL of the lysates or of the medium were diluted 1:3 in the assay buffer (0.2 mM 3-(2-hydroxyphenyl) propionic acid, 10 mM *p*-Nitrobenzene diazonium fluoroborate solution, 0.4 M citrate buffer, pH 3.5, at 1:1:2 volume). The samples were incubated 5 min at 37 °C degree and resolved by an HPLC system equipped with a UV detector (SPD-20A) (Shimadzu, Kyoto, Japan), using the elution conditions described in [21]. The amount of [^3^H]-acetoacetate, an index of KBs synthesized by [^3^H]-acetate, was quantified according to calibration curves of serial dilutions of acetoacetate and expressed as µmol/mg cell proteins.

### 2.13. TCA Cycle Enzymes Activity

The enzymatic activities of citrate synthase, α-ketoglutarate dehydrogenase (DH), succinate dehydrogenase, and malate dehydrogenase were measured on 10 mg mitochondrial proteins using the Citrate Synthase Assay Kit (Sigma-Aldrich, St. Louis, MO, USA, catalog n° MAK193), Alpha Ketoglutarate (alpha KG) Assay Kit (Abcam, Cambridge, UK, catalog n° ab83431), Malate Dehydrogenase Assay Kit (Sigma-Aldrich, St. Louis, MO, USA, catalog n° MAK196), Succinate Dehydrogenase Activity Colorimetric Assay Kit (BioVision, Milpitas, CA, USA, catalog n° K660), as per manufacturer’s instructions. Results were expressed as mU/mg mitochondrial proteins.

### 2.14. The Activity of Mitochondrial ETC Complexes I–IV

According to [22], cells were washed twice in ice-cold 0.1 M phosphate-buffered saline (PBS), then lysed in 0.5 mL buffer A (50 mmol/L Tris, 100 mmol/L KCl, 5 mmol/L MgCl_2_, 1.8 mmol/L ATP, 1 mmol/L EDTA, pH 7.2), supplemented with protease-inhibitor cocktail III [100 mmol/L AEBSF, 80 mmol/L aprotinin, 5 mmol/L bestatin, 1.5 mmol/L E-64, 2 mmol/L leupeptin and 1 mmol/L pepstatin (Merck, Darmstadt, Germany), 1 mmol/L phenylmethylsulfonyl fluoride (PMSF), 250 mmol/L NaF. Samples were clarified by centrifuging at 650× *g* for 3 min at 4 °C, and the supernatant was collected and centrifuged at 13,000× *g* for 5 min at 4 °C. The new supernatant was discarded and the pellet containing mitochondria was washed in 0.5 mL buffer A and re-suspended in 0.25 mL buffer B (250 mmol/L sucrose, 15 μmol/L K_2_HPO_4_, 2 mmol/L MgCl_2_, 0.5 mmol/L EDTA, 5% *w*/*v* bovine serum albumin). A 100 μL aliquot was sonicated and used for the measurement of protein content. The remaining not-sonicated part was used to measure the electron transport chain (ETC) complexes I–IV activities according to [22]. Results were expressed as nmol NAD+/min/mg mitochondrial protein for complex I, nmol cyt c reduced/min/mg mitochondrial protein for complexes II–III, and nmoles cyt c oxidized/min/mg mitochondrial protein for complex IV.

### 2.15. ATP Levels in Mitochondria

The ATP levels in mitochondria extracts were measured with the ATP Bioluminescent Assay Kit (Sigma-Aldrich, St. Louis, MO, USA). ATP was quantified as relative light units (RLU) and converted into nmoles ATP/mg mitochondrial proteins, according to the calibration curve previously set.

### 2.16. Statistics

Statistical comparisons were conducted in Prism (GraphPad Software, Inc., La Jolla, CA, USA). Results are expressed as mean ± SEM.

## 3. Results

### 3.1. FLVCR1a Is Highly Expressed in Tumor-Associated Endothelial Cells (TECs)

**Figure 1:**
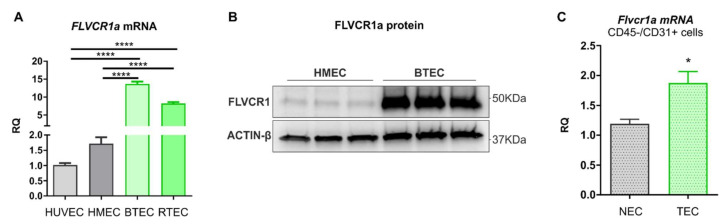
FLVCR1a expression in healthy and tumor endothelial cells (ECs). (**A**) qRT-PCR analysis showing FLVCR1a mRNA levels in human ECs. (**B**) Western blot analysis showing FLVCR1a protein expression in healthy (HMEC) and tumor-derived (BTEC) human ECs. (**C**) qRT-PCR analysis showing FLVCR1a mRNA levels in mouse ECs. Murine ECs were isolated from tumors (TEC) or lungs (NEC) through magnetic separation using a CD45 negative selection followed by a CD31 positive selection. HUVEC, Human Umbilical Endothelial Cells; HMEC, human dermal microvascular endothelial cells; BTEC, breast-tumor-derived endothelial Cells; RTEC, renal carcinoma endothelial cells; NEC/TEC, normal/tumor endothelial cells; RQ, relative quantification. Data are representative of at least 3 independent experiments and are expressed as mean ± SEM of *n* = 3 (**A**) or *n* = 4 (**C**). * *p* < 0.05, **** *p* < 0.0001; For statistical analyses, an ordinary one-way ANOVA test with Tukey’s multiple comparisons (**A**) and a nonparametric Mann–Whitney test (**C**) were used.

The tumor vasculature differs from the normal vascular beds found in healthy organs in multiple aspects. This vascular heterogeneity resides at least in part in specific gene expression profiles that ultimately shape most of the architecture and function of the vasculature itself. To elucidate the role of heme metabolism in the vascular system, the expression of the heme exporter *FLVCR1a* was evaluated in different types of human ECs deriving from both normal and tumor tissues. For this purpose, two different lines of normal ECs (NECs) were used, which are macrovascular ECs from the human umbilical vein (HUVECs) and microvascular ECs from the human dermis (HMECs). For the tumor-associated endothelial counterpart, breast tumor-derived ECs (BTECs) and renal carcinoma-derived ECs (RTECs) were analyzed. As shown in Figure 1A,B, *FLVCR1a* expression was increased in human TECs compared to NECs. To confirm this result in a more reliable system, LLC cells were subcutaneously injected into the flanks of syngeneic wild-type mice, thus forming ectopic tumors. After 14 days, murine TECs and NECs were isolated from tumors and lungs, respectively. As reported in Figure 1C, *Flvcr1a* transcript was upregulated in TEC compared to NEC in vivo. These data, taken together, suggest that TECs are highly dependent on FLVCR1a expression to accomplish their function.

### 3.2. FLVCR1a-Deficient TECs Show Increased FAO, Ketogenesis, and Ketone Bodies (KBs) Secretion

**Figure 2:**
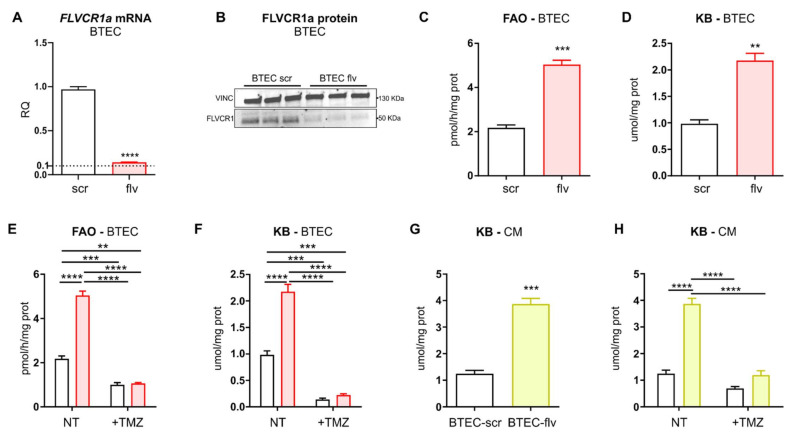
Metabolic changes found in human BTECs upon *FLVCR1a* silencing. (**A**,**B**) The mRNA (**A**) and protein (**B**) levels of *FLVCR1a* are shown. Data are representative of at least 3 independent experiments and are expressed as mean ± SEM. ** *p* < 0.01. (**C**) Fatty acids β-oxidation (FAO) in *FLVCR1a*-silenced (flv) and control (scr, i.e., “scramble shRNA”) BTECs. Values are expressed as pmol of 14C-acid-soluble metabolites (14C-ASM)/h/mg of proteins. Data represent means ± SEM of *n* = 3. For statistical analyses, an unpaired Student’s *t*-test was used. *** *p* < 0.001. (**D**) β-hydroxybutyrate (β-OH) levels expressed as µmol/mg of cellular proteins. The measure of β-OH was considered as an index of ketone bodies (KBs) amount. Data represent means ± SEM, *n* = 3. For statistical analyses, an unpaired Student’s *t*-test was used. ** *p* < 0.01. (**E**,**F**) FAO and β-OH levels in *FLVCR1a*-silenced (red) and control (white) BTECs, under basal conditions or upon treatment with the FAO inhibitor trimetazidine (TMZ, 500 μM). Data represent means ± SEM, *n* = 3. For statistical analyses, a two-way ANOVA test with Tukey’s multiple comparisons was used. ** *p* < 0.01, *** *p* < 0.001, **** *p* < 0.0001. (**G**,**H**) β-OH levels in culture medium collected after 72 h of *FLVCR1a*-silenced (yellow) and control (white) BTECs culture, (**G**) under basal conditions or (**H**) upon treatment with 500 μM TMZ. Data represent means ± SEM, *n* = 3. For statistical analyses, an unpaired Student’s *t*-test (**G**) and two-way ANOVA test with Tukey’s multiple comparisons (**H**) were used. *** *p* < 0.001, **** *p* < 0.0001.

To dissect the role of heme metabolism in human TECs, endothelial heme homeostasis was disrupted in BTECs by silencing the heme exporter FLVCR1a (Figure 2A,B). It has been previously shown that the heme synthesis–export axis controls TCA-cycle fuelling in CCs [16]. Moreover, FLVCR1a-targeting in BTECs results in enhanced TCA cycle flux [16]. For this reason, metabolic pathways interlinked with the TCA cycle have been evaluated in *FLVCR1a*-silenced BTECs. Interestingly, a strongly increased FAO was detected in *FLVCR1a*-silenced BTECs (Figure 2C). The end product of FAO is acetyl-CoA, a crucial metabolite that, besides entering the TCA cycle, can generate KBs. Accordingly, the amount of KBs was increased in *FLVCR1a*-silenced BTECs, as demonstrated by the higher amount of β-hydroxybutyrate (β-OH) (Figure 2D). To demonstrate that the KBs present in *FLVCR1a*-silenced BTECs were produced by FAO-derived acetyl-CoA, KBs measurement was repeated in the presence of trimetazidine (TMZ), an FAO inhibitor (Figure 2E). FAO inhibition was sufficient to abolish the increase in ketogenesis observed in *FLVCR1a*-silenced BTECs (Figure 2F).

To investigate if EC-derived KBs were released in the extracellular environment, the levels of β-OH were measured in the medium after 72 h of cell culture. Importantly, KBs were highly enriched in the conditioned medium (CM) collected from *FLVCR1a*-silenced BTECs (Figure 2G). Furthermore, KBs’ accumulation in *FLVCR1a*-silenced BTECs-derived CM was completely abrogated following FAO inhibition in BTECs (Figure 2H).

**Figure 3:**
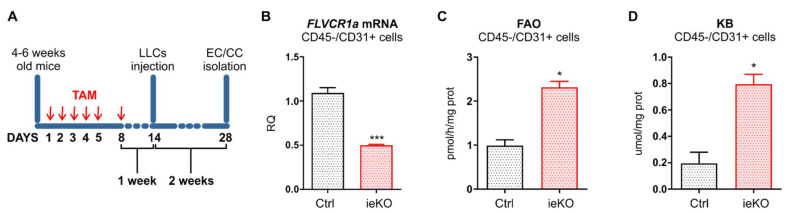
Metabolic changes found in TECs isolated from LLC-xenografts developed into *Flvcr1a*^iEC-KO^ mice. (**A**) Schematic representation of tamoxifen (TAM) treatment in *Flvcr1a*^flox/flox^; Cdh5(PAC)-Cre^ERT2^ and *Flvcr1a*^flox/flox^ control mice. Briefly, 4–6 weeks old mice were treated intraperitoneally with 1 mg/day tamoxifen for 5 consecutive days, followed by 1 additional day after a 2-day treatment-free interval (red arrows). After one week after TAM injection, 5 × 10^5^ LLC cells suspended in 100 μL PBS were injected subcutaneously into the mice flanks. Tumors were dissected after 12–14 days from LLC cells injection. (**B**) qRT-PCR analysis showing *Flvcr1a* mRNA levels in CD45−/CD31+ cells isolated from LLC xenografts developed in *Flvcr1a*^iEC-KO^ (ieKO) and control (Ctrl) mice. Data represent means ± SEM, *n* = 3. For statistical analyses, an unpaired Student’s *t*-test was used. *** *p* < 0.001. (**C**,**D**) FAO and β-OH levels measured in CD45−/CD31+ cells isolated from LLC xenografts developed in *Flvcr1a*^iEC-KO^ (ieKO) and control (Ctrl) mice. FAO values are expressed as pmol of 14C-acid-soluble metabolites (14C-ASM)/h/mg of proteins. β-OH levels are expressed as µmol/mg of cellular proteins. The measure of β-OH was considered as an index of KBs amount. Data represent means ± SEM, *n* = 2 pools of animals. For statistical analyses, an unpaired Student’s *t*-test was used. * *p* < 0.05. EC, endothelial cell; CC, cancer cell; FAO, fatty acid β-oxidation; KB, ketone body.

To inhibit *Flvcr1a* in vivo, a genetic approach was used. Cdh5(PAC)-Cre^ERT2^ mice were crossed with *Flvcr1a*^flox/flox^ mice, and 4–6 weeks old progenies were treated with tamoxifen to obtain *Flvcr1a*^∆EC/∆EC^ mice, deficient for *Flvcr1a* in ECs (*Flvcr1a*^iEC-KO^). One week after tamoxifen treatment, LLC tumor cells were injected into the flanks of *Flvcr1a*^iEC-KO^ syngeneic mice, and 14 days later, the tumors were dissected, and TECs were isolated through magnetic separation with a microbeads-conjugated CD31 antibody (Figure 3A). To assess the efficacy of gene deletion, *Flvcr1a* expression was evaluated by performing quantitative real-time PCR (qRT-PCR) on TECs isolated from subcutaneous tumors. This analysis revealed that *Flvcr1a* expression was reduced by about 50–60% in TECs isolated from *Flvcr1a*^iEC-KO^ mice compared to those derived from control mice (Figure 3B). Similar to what was observed in human BTECs, FAO and KBs levels were strongly enhanced in TECs lacking *Flvcr1a* in vivo (Figure 3C,D).

Taken together, these data indicate that heme export disruption enhances FAO in TECs, resulting in increased production and secretion of KBs in the extracellular environment.

### 3.3. FLVCR1a Silencing in TECs Induces a Metabolic Shift towards OXPHOS in Cancer Cells (CCs)

**Figure 4:**
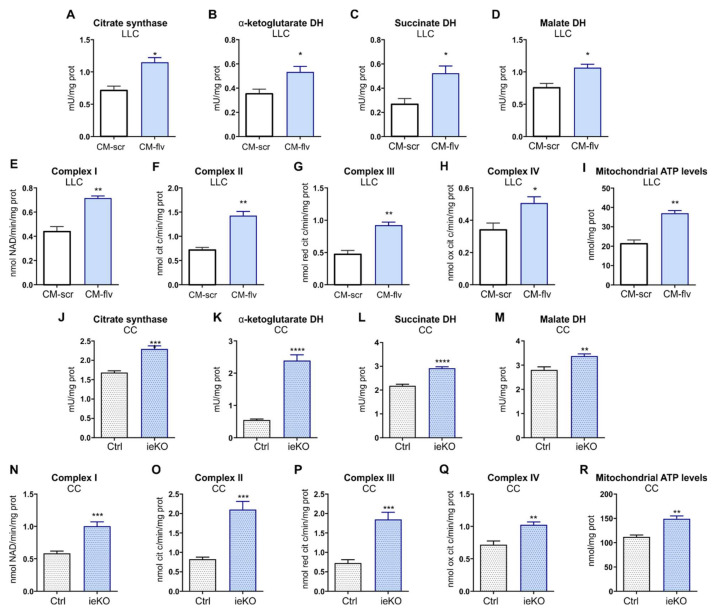
FLVCR1a deficiency in TECs induces a metabolic rewiring in cancer cells (CCs). (**A**–**H**) Activities of TCA cycle enzymes citrate synthase, α-ketoglutarate dehydrogenase (DH), succinate DH and malate DH, and the mitochondrial electron transport chain (ETC) complexes I–IV in LLC cells treated for 24 h with *FLVCR1a*-silenced (CM-flv) or control (CM-scr) BTECs-derived CM. Results are expressed as mU/mg mitochondrial proteins for TCA enzymes, nmol NAD+/min/mg of mitochondrial protein for complex I, nmol reduced cytochrome c/min/mg of mitochondrial protein for complexes II–III, and nmol oxidized cytochrome c/min/mg of mitochondrial protein for complex IV. Data represent means ± SEM, *n* = 3 wells. For statistical analyses, an unpaired Student’s *t*-test was used. * *p* < 0.05, ** *p* < 0.01. (**I**) Mitochondrial ATP levels were measured with a bioluminescent assay kit in LLC cells treated for 24 h with *FLVCR1a*-silenced (CM-flv) or control (CM-scr) BTECs-derived CM. Results are expressed as nmol/mg of mitochondrial proteins. Data represent means ± SEM, *n* = 3 wells. For statistical analyses, an unpaired Student’s *t*-test was used. ** *p* < 0.01. (**J**–**Q**) Activities of TCA cycle enzymes citrate synthase, α-ketoglutarate DH, succinate DH and malate DH, and mitochondrial ETC complexes I–IV in CCs isolated by LLC-xenografts developed in *Flvcr1a*^iEC-KO^ (ieKO) and control (Ctrl) mice. Data are expressed as mU/mg mitochondrial proteins for TCA enzymes, nmol NAD+/min/mg of mitochondrial protein for complex I, nmol reduced cytochrome c/min/mg of mitochondrial protein for complexes II–III, and nmol oxidized cytochrome c/min/mg of mitochondrial protein for complex IV. Data represent means ± SEM, *n* = 5. For statistical analyses, an unpaired Student’s *t*-test was used. ** *p* < 0.01, *** *p* < 0.001. **** *p* < 0.0001. (**R**) Mitochondrial ATP levels were measured with a bioluminescent assay kit in CCs isolated by LLC-xenografts developed in *Flvcr1a*^iEC-KO^ (ieKO) and control (Ctrl) mice. Results are expressed as nmol/mg of mitochondrial proteins. Data represent means ± SEM, *n* = 5. For statistical analyses, an unpaired Student’s *t*-test was used. ** *p* < 0.01.

To investigate whether metabolic alterations occurring in TECs upon *FLVCR1a* silencing had an impact on CCs, LLC cells metabolism was assessed following 24 h treatment with CM derived from *FLVCR1a*-silenced BTECs and control cells. Notably, the activity of the first enzyme of the TCA-citrate synthase and of other three oxido-reductive TCA enzymes (α-ketoglutarate dehydrogenase, succinate dehydrogenase, malate dehydrogenase), the electron flux through ETC complexes and the levels of mitochondrial ATP were increased in LLC cells treated with *FLVCR1a*-silenced BTECs CM (Figure 4A–I). Accordingly, similar results were obtained in vivo when comparing CCs isolated from LLC-derived xenografts generated in *Flvcr1a*^iEC-KO^ and control mice (Figure 4J–R).

These data indicate that FLVCR1a-targeting in TECs promotes a metabolic rewiring in the CC, enhancing the TCA cycle and oxidative metabolism.

**Figure 5:**
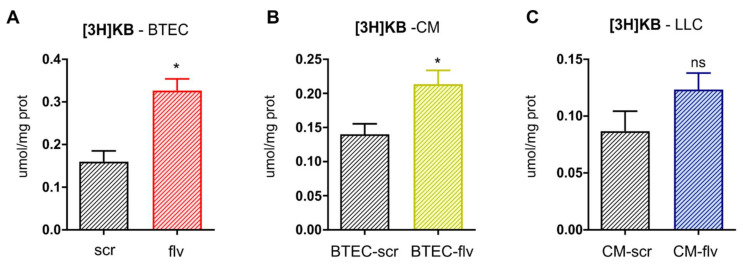
KBs derived from FLVCR1a-deficient TEC can be transferred to the CC. (**A**–**C**) Quantification of [^3^H]-acetoacetate by high-performance liquid chromatography (HPLC) (**A**) in *FLVCR1a*-silenced (flv) and control (scr) BTECs lysates, (**B**) in *FLVCR1a*-silenced (BTEC-flv) and control (BTEC-scr) BTECs-derived CM collected after 72 h of cell culture, and (**C**) in LLC cells lysates after 24 h of conditioning with *FLVCR1a*-silenced (CM-flv) and control (CM-scr) BTECs-derived CM. Data are expressed as µmol/mg cell proteins and represent means ± SEM, *n* = 3. For statistical analyses, an unpaired Student’s *t*-test was used. * *p* < 0.05; ns, not significant.

An increased concentration of KBs within the TME may alter energy fuel selection by the CC, attenuating glucose utilization in favor of KBs oxidation, which in turn can promote an oxidative metabolism by sustaining the TCA cycle flux. Since *FLVCR1a*-silenced BTECs showed a higher production and secretion of KBs, tracing experiments were performed to assess whether BTEC-derived KBs can be transferred to LLC cells and contribute to their metabolic rearrangement. For this purpose, BTECs were labeled with [^3^H]-acetate, i.e., the FAO product that may serve as a precursor for KBs as acetoacetate and β-hydroxybutyrate. The amount of [^3^H]-acetoacetate was then measured by HPLC in BTECs lysates as well as in the CM after 72 h. In line with previous results, tracing experiments confirmed that, upon FLVCR1a-targeting, BTECs showed enhanced incorporation of [^3^H]-acetate into [^3^H]-acetoacetate, suggesting an endogenous generation of KBs from acetate within BTEC (Figure 5A). Moreover, a higher secretion of the radiolabeled KB was detected in the extracellular medium (Figure 5B). Finally, [^3^H]-acetoacetate was detected in LLC cells treated with CM for 24 h, indicating that TEC-derived KBs can be taken up by CCs (Figure 5C).

**Figure 6:**
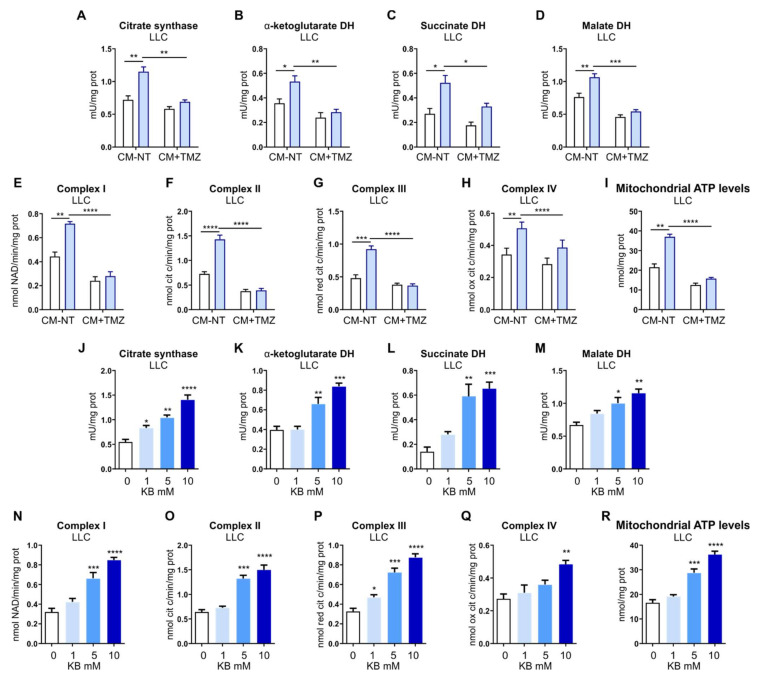
Endothelial-derived KBs are involved in CCs metabolic rewiring. (**A**–**H**) Activities of TCA cycle enzymes citrate synthase, α-ketoglutarate DH, succinate DH and malate DH, and mitochondrial ETC complexes I–IV in LLC cells treated for 24 h with *FLVCR1a*-silenced (light blue) or control (white) BTECs-derived CM, under basal conditions (CM-NT) or following BTECs treatment with 500 μM trimetazidine (CM + TMZ). Data are expressed as mU/mg mitochondrial proteins for TCA enzymes, nmol NAD+/min/mg of mitochondrial protein for complex I, nmol reduced cytochrome c/min/mg of mitochondrial protein for complexes II–III, and nmol oxidized cytochrome c/min/mg of mitochondrial protein for complex IV. Data represent means ± SEM, *n* = 3. For statistical analyses, two-way ANOVA test with Tukey’s multiple comparisons was used. * *p* < 0.05, ** *p* < 0.01, *** *p* < 0.001, **** *p* < 0.0001. (**I**) Mitochondrial ATP levels were measured with a bioluminescent assay kit in LLC cells treated for 24 h with *FLVCR1a*-silenced (light blue) or control (white) BTECs-derived CM, under basal conditions (CM-NT) or following BTECs treatment with 500 μM trimetazidine (CM + TMZ). Results are expressed as nmol/mg of mitochondrial proteins. Data represent means ± SEM, *n* = 3. For statistical analyses, two-way ANOVA test with Tukey’s multiple comparisons was used. ** *p* < 0.01, **** *p* < 0.0001. (**J**–**Q**) Activities of TCA cycle enzymes citrate synthase, α-ketoglutarate DH, succinate DH and malate DH, and mitochondrial ETC complexes I–IV in LLC cells treated for 24 h with increasing concentration of β-OH, i.e., 1, 5 and 10 mM. Data are expressed as mU/mg mitochondrial proteins for TCA enzymes, nmol NAD+/min/mg of mitochondrial protein for complex I, nmol reduced cytochrome c/min/mg of mitochondrial protein for complexes II–III, and nmol oxidized cytochrome c/min/mg of mitochondrial protein for complex IV. Data represent means ± SEM, *n* = 3. For statistical analyses, an ordinary one-way ANOVA test with Dunnett’s multiple comparisons test was used. * *p* < 0.05, ** *p* < 0.01, *** *p* < 0.001, **** *p* < 0.0001. (**R**) Mitochondrial ATP levels were measured with a bioluminescent assay kit in LLC cells treated for 24 h with increasing concentrations of β-OH, i.e., 1, 5, and 10 mM. Results are expressed as nmol/mg of mitochondrial proteins. Data represent means ± SEM, *n* = 3. For statistical analyses, an ordinary one-way ANOVA test with Dunnett’s multiple comparisons was used. *** *p* < 0.001, **** *p* < 0.0001.

To understand whether BTEC-derived KBs are involved in the metabolic shift observed in CCs following CM treatment, FAO was inhibited in BTECs, and the resulting CM was used to treat LLC cells. As shown in Figure 6A–I, the inhibition of FAO in BTECs was sufficient to prevent the induction of TCA- and OXPHOS-based metabolism in LLC cells. These data, taken together, indicate that *FLVCR1a*-silenced BTECs-derived KBs can trigger a metabolic shift in LLC cells by sustaining the TCA cycle and OXPHOS. To further support this hypothesis, LLC cells were treated with increasing concentrations of β-OH (i.e., 1 mM, 5 mM, and 10 mM). As shown in Figure 6J–M, β-OH was able to significantly increase the activity of TCA enzymes, ETC complexes I, II, and III. Moreover, increased activity of complex IV was found with 10 mM β-OH (Figure 6N–Q). In keeping with these data, a higher mitochondrial ATP level was found in LLC cells treated with 5 or 10 mM β-OH (Figure 6R).

These experiments demonstrate that KBs’ enrichment in the extracellular environment enhances the TCA cycle activity and the oxidative metabolism in CCs, thus resembling the effects observed using the *FLVCR1a*-silenced BTECs-derived CM.

## 4. Discussion

The results described here provide evidence of heme metabolism involvement in the crosstalk between tumor-associated endothelial cells (TECs) and cancer cells (CCs). The active role of the tumor microenvironment (TME) in cancer development and progression has become increasingly recognized [23]. One of the most studied interplays within the TME involves endothelial cells (ECs) and CCs. Since blood vessels are required to supply oxygen and nutrients to the tumor, CCs stimulate angiogenesis by secreting growth factors, such as the Vascular-Endothelial Growth Factor (VEGF), which triggers EC re-activation. Nevertheless, the landscape is more complex than that, and metabolism plays a prominent role [24]. CCs are indeed able to metabolically hijack and induce reprogramming in the surrounding stromal cells, including ECs. Consistently, TEC metabolism is profoundly perturbed in cancer [25,26]. For instance, the glycolytic rate is strongly increased in active angiogenic TECs compared to healthy ECs, thus sustaining the enhanced motility and active proliferation [27]. Basically, TECs display a specific metabolic profile, which underlies a plethora of specialized endothelial functions that favor CC proliferation and support tumor growth. Moreover, the crosstalk is bidirectional, and increasing evidence highlights the importance of the metabolic cooperation that is established between CCs and surrounding stromal cells [5].

**Figure 7:**
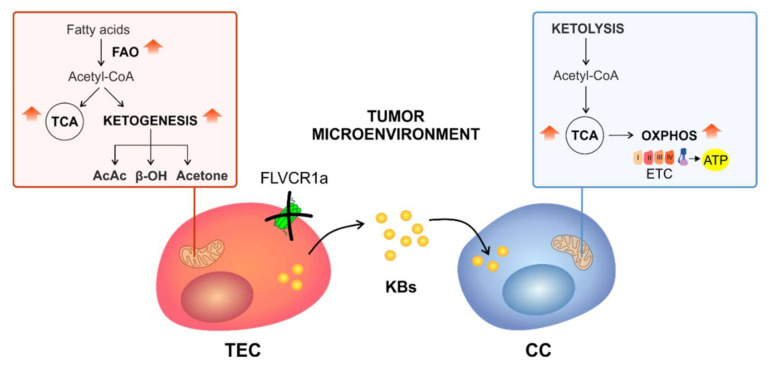
Targeting heme metabolism in TECs induces a metabolic shift in CCs. In the proposed model, the loss of the cell membrane heme-exporter FLVCR1a in TECs results in increased TCA cycle fuelling by FAO. Due to the high rate of FAO, FLVCR1a-deficient TECs also display KBs accumulation, which can be secreted in the TME. Here, KBs are taken up by surrounding CCs, where they promote mitochondrial oxidative metabolism (OXPHOS). TEC, tumor endothelial cell; CC, cancer cell; FAO, fatty acid β-oxidation; TCA, tricarboxylic acid cycle; KBs, ketone bodies; AcAc, acetoacetate; β-OH, β-hydroxybutyrate; ETC, electron transport chain; ATP, adenosine triphosphate; OXPHOS, oxidative phosphorylation; red arrows, increased activity of the metabolic pathways.

Heme is a vital molecule involved in several biological processes, and recent findings pointed out its crucial role in ECs, specifically in the regulation of energy metabolism and angiogenesis [16,19,28]. In particular, the loss of the cell membrane heme exporter FLVCR1a in ECs leads to embryonic death associated with impaired angiogenesis [19]. Moreover, it has been recently demonstrated that the heme synthesis–export system is involved in the regulation of TEC energy metabolism. Indeed, FLVCR1a deficiency in active TECs results in increased TCA cycle flux and OXPHOS both in vitro and in vivo [16]. Here, we found that FLVCR1a-deficient TECs enhanced FAO, which likely sustained the TCA cycle by providing acetyl-CoA (Figure 7). Interestingly, FAs-derived acetyl-CoA can also be incorporated into KBs, small molecules that are produced through ketogenesis. KBs, namely, acetoacetate (AcAc), β-hydroxybutyrate (β-OHB), and acetone, represent an alternative energy source to glucose and are primarily synthetized in the liver under conditions of glucose shortage such as during fasting or prolonged exercise [29]. Noteworthy, we observed an accumulation of KBs in FLVCR1a-deficient TECs (Figure 7). This result agrees with our previous work showing higher FAO activity as well as increased β-OHB levels in *FLVCR1a*-silenced colorectal cancer cells [16]. Ketogenesis consists of multiple reactions occurring in mitochondria. First, two acetyl-CoA are condensed to form acetoacetyl-CoA (AcAc-CoA). A third acetyl-CoA is then added to produce β-hydroxy-β-methylglutaryl-CoA (HMG-CoA), a reaction catalyzed by the rate-limiting enzyme HMG-CoA synthase 2 (HMGCS2). Finally, the activity of β-hydroxymethyl-β-methylglutaryl-CoA lyase (HMGCL) generates AcAc that can spontaneously decarboxylate to acetone or be further converted into β-OHB by β-OHB dehydrogenase 1 (BDH1). While acetone can easily diffuse across cell membranes, AcAc and β-OHB are mainly transported via monocarboxylate transporters (in mammals, MCT1 and MCT2). As an alternative source of energy, circulating KBs are oxidized in extrahepatic tissues such as the heart, skeletal muscle, kidney, and brain to fuel mitochondrial metabolism in conditions of low glucose availability. KBs oxidation (ketolysis) requires the enzyme succinyl-CoA:3-oxoacid-CoA transferase (SCOT/OXCT1), which activates AcAc to AcAc-CoA through the exchange of a CoA moiety from succinyl-CoA [29]. Importantly, succinyl-CoA is a hub metabolite shared across the TCA cycle, KBs oxidation, and heme biosynthesis. Hence, succinyl-CoA consumption through ketolysis might affect heme metabolism and vice versa. Finally, a reversible AcAc-CoA thiolase reaction yields two molecules of acetyl-CoA, which enter the TCA cycle and sustain oxidative metabolism.

The mechanism behind the KBs-mediated oxidative metabolic shift found in CCs conditioned by *FLVCR1a*-deficient ECs is not fully elucidated. Tracing experiments with labeled acetate (i.e., [^3^H]-acetate) supplementation in ECs clearly demonstrated that LLC cells are capable of importing KBs from the extracellular environment. In this context, monocarboxylate transporters (MCTs) may play a relevant role. Indeed, MCTs are involved in KBs transport across cell membranes and are generally dysregulated in cancer [30]. Despite the enrichment of labeled KBs found in *FLVCR1a*-silenced BTECs-derived CM following [^3^H]-acetate supplementation, conditioned LLC cells showed only a slight increase in labeled KBs intracellular content. This result supports the hypothesis of rapid utilization of KBs to fuel the TCA cycle once taken up by the cancer cell.

Nevertheless, recent evidence also attributes to KBs a signaling activity. For instance, β-OHB is an endogenous inhibitor of class I nuclear histone deacetylase (HDACs) enzymes (i.e., HDAC1, HDAC3, HDAC4) in a dose-dependent manner (IC50 ~2–5 mM). Consistently, KBs enrichment in the extracellular milieu promotes histone hyperacetylation, resulting in a modified epigenetic landscape and subsequent rewired gene expression profile [31,32]. Although histones were the first identified targets, many non-histone proteins are subjected to HDACs-mediated deacetylation, including the proto-oncogene c-Myc and the tumor suppressor p53. Moreover, KBs may also indirectly contribute to protein hyperacetylation by increasing the intracellular pool of acetyl-CoA [33].

β-OHB is the ligand for at least two G-protein-coupled receptors (GPRs or GPCRs) that bind short-chain fatty acids (FAs). GPR109A, even known as hydroxycarboxylic acid receptor 2 (HCAR2), is a Gi/o coupled GPCR showing an intermediate affinity for β-OHB (EC50 ~0.8 mM). Its activation reduces lipolysis in adipocytes, probably representing a feedback mechanism to regulate FA availability for KBs metabolism. Interestingly, GPR109A was recently identified as a tumor suppressor [33]. Indeed, colon CCs express GPR109A at very low levels, whereas breast cancer cells completely abolish its expression. Noteworthy, GPR109A ectopic expression in colon CCs followed by GPR109A agonist exposure exerted anti-tumoral activity by increasing CC apoptosis [34]. Furthermore, β-OHB is the antagonist of the GPR81 l-lactate receptor, also known as HCAR1. GPR81 is widely expressed in tissues, including ECs, and physiologically promotes angiogenesis upon exercise-associated increased circulating lactate. Consistent with the definition of lactate as an oncometabolite, GPR81 is overexpressed in many cancers, and in most cases, its expression levels positively correlate with tumor growth and metastasis. Indeed, lactate accumulates within the TME, promoting angiogenesis and shaping a pro-tumoral niche [34,35]. GPR81 activation results in decreased intracellular cyclic AMP (cAMP) and increased cellular Ca2+ levels, ultimately promoting CC growth. In such a context, β-OHB could antagonize the pro-tumorigenic effects of lactate by interacting with GPR81 [34,35]. Interestingly, a recent study points out a vasodilator effect of β-OHB on the vasculature, primarily mediated by potassium channels and independent of the traditional GPCRs activity [36]. Hence, it would be interesting to understand whether in FLVCR1a deficiency models, KBs, besides altering the metabolism of surrounding CCs, might also impact the tumor vasculature architecture.

The connection between KBs and cancer is increasingly recognized. Nevertheless, current knowledge suggests a tumor-type-specific ability to use KBs as an alternative energetic fuel. Unlike normal cells, several CCs are not able to shift towards KB oxidation to sustain energetic metabolism. Consistently, ketolytic enzyme expression levels, such as BDH1 and SCOT, are low or undetectable in pancreatic cancer (PANC-1), lung cancer (H1299), and neuroblastoma (SK-N-AS) human cell lines as well as in human glioblastoma in vivo [32,37]. Consistently, KBs treatment inhibits neuroblastoma cell viability in vitro, whereas combination therapy of a ketogenic diet (KD) and antiangiogenic drug decreases tumor growth in a glioblastoma mouse model [38,39]. Furthermore, KD alone reduces tumor burden in a mouse model of metastatic brain cancer [40]. Similarly, KD administration in mice significantly inhibits liver cancer cell growth [41]. Finally, a recent study shows that β-OHB delays growth of melanoma orthotopic tumors by acting on the host immune system and displays synergistic antitumorigenic effects when given in combination with immunotherapy [42]. Different outcomes come from studies on breast cancer. β-OHB administration in mice carrying MDA-MB-231 breast cancer cells xenografts results in a 2.5-fold increase in tumor volume [43]. Moreover, ketogenic fibroblasts overexpressing HMGCS2 enhance the growth of co-injected MDA-MB-231 breast cancer cells in vivo [44]. These data suggest that, differently from other cancer cell types, breast cancer cells can utilize fibroblast-derived KBs to fuel oxidative mitochondrial metabolism and benefit from this metabolic coupling. In spontaneous lung cancers, KBs are known sources of energy for cancer cells: interestingly, the switch from an anaerobic-glycolysis-dependent metabolism toward an FAO- and KB-oxidation-prevailing metabolism depends on the micro-environmental changes induced by treatment with antiangiogenic drugs [45]. This metabolic rewiring allows sustained tumor growth over the long term despite exposure to antiangiogenic drugs but makes the tumor dependent on mitochondrial metabolism. In this scenario, the co-treatment with both antiangiogenics and mitochondrial metabolism inhibitors abrogates tumor growth owing to a metabolic synthetic lethality [45]. Based on this evidence, FLVCR1a-targeting in tumor-associated ECs could unveil a metabolic vulnerability by making the tumor dependent on mitochondrial metabolism. In this scenario, combinatorial treatment with mitochondrial-respiration inhibitors and agents targeting heme metabolism might be a promising strategy for cancer treatment.

## 5. Conclusions

In the present study, we show that FLVCR1a-deficient TECs are able to shape metabolism in CCs (Figure 7). In particular, LLC cells treated with FLVCR1a-silenced BTECs-derived CM displayed enhanced TCA cycle enzymes activity and increased oxidative metabolism. The metabolic rewiring observed in CCs upon CM-treatment was likely due to the high concentration of KBs found in FLVCR1a-silenced BTECs-derived CM. Indeed, KBs treatment alone in LLC cells completely resembled the effects of FLVCR1a-silenced, BTECs-derived CM. These data suggest that, by modulating the TME, TEC heme metabolism forces the surrounding CCs to acquire a similar energetic metabolic profile. Importantly, this metabolic shift might affect CCs viability or migratory potential. In the future, it would be interesting to investigate the impact of FLVCR1a-deficient TECs on primary tumor growth as well as on the process of tumor metastatization. Finally, further studies are required to elucidate the interplay between heme and KBs metabolism.

Great efforts are being made in order to identify new metabolic vulnerabilities within the TME that can be exploited in cancer treatment. In this perspective, targeting heme metabolism in TECs opens new opportunities to reshape the TME.

## 6. Patents

The University of Turin filed a patent application (n° 102021000015368_11 June 2021), whose inventors are A.L.A., F.B., S.P., D.C., V.F., and E.T., with data included in this manuscript.

## Data Availability

The data presented in this study are available on request from the corresponding author.
